# Longitudinal Reduction in Diversity of Maternal Gut Microbiota During Pregnancy Is Observed in Multiple Low-Resource Settings: Results From the Women First Trial

**DOI:** 10.3389/fmicb.2022.823757

**Published:** 2022-08-01

**Authors:** Minghua Tang, Nicholas E. Weaver, Daniel N. Frank, Diana Ir, Charles E. Robertson, Jennifer F. Kemp, Jamie Westcott, Kartik Shankar, Ana L. Garces, Lester Figueroa, Antoinette K. Tshefu, Adrien L. Lokangaka, Shivaprasad S. Goudar, Manjunath Somannavar, Sumera Aziz, Sarah Saleem, Elizabeth M. McClure, K. Michael Hambidge, Audrey E. Hendricks, Nancy F. Krebs

**Affiliations:** ^1^Department of Pediatrics, Section of Nutrition, University of Colorado Anschutz Medical Campus, Aurora, CO, United States; ^2^Department of Mathematical and Statistical Sciences, University of Colorado, Denver, Denver, CO, United States; ^3^Department of Infectious Disease, University of Colorado Anschutz Medical Campus, Aurora, CO, United States; ^4^Institute of Nutrition in Central America and Panama (INCAP), Guatemala City, Guatemala; ^5^KLE Academy of Higher Education and Research (Deemed-to-be-University), Jawaharlal Nehru Medical College, Belagavi, India; ^6^Department of Community Health Sciences, Aga Khan University, Karachi, Pakistan; ^7^RTI International, Durham, NC, United States

**Keywords:** low middle income countries, gut microbiota, inflammation, pregnancy, small quantity lipid-based nutrient supplements (SQ-LNS)

## Abstract

**Objective:**

To characterize the changes in gut microbiota during pregnancy and determine the effects of nutritional intervention on gut microbiota in women from sub-Saharan Africa (the Democratic Republic of the Congo, DRC), South Asia (India and Pakistan), and Central America (Guatemala).

**Methods:**

Pregnant women in the Women First (WF) Preconception Maternal Nutrition Trial were included in this analysis. Participants were randomized to receive a lipid-based micronutrient supplement either ≥3 months before pregnancy (Arm 1); started the same intervention late in the first trimester (Arm 2); or received no nutrition supplements besides those self-administered or prescribed through local health services (Arm 3). Stool and blood samples were collected during the first and third trimesters. Findings presented here include fecal 16S rRNA gene-based profiling and systemic and intestinal inflammatory biomarkers, including alpha (1)-acid glycoprotein (AGP), C-reactive protein (CRP), fecal myeloperoxidase (MPO), and calprotectin.

**Results:**

Stool samples were collected from 640 women (DRC, *n* = 157; India, *n* = 102; Guatemala, *n* = 276; and Pakistan, *n* = 105). Gut microbial community structure did not differ by intervention arm but changed significantly during pregnancy. Richness, a measure of alpha-diversity, decreased over pregnancy. Community composition (beta-diversity) also showed a significant change from first to third trimester in all four sites. Of the top 10 most abundant genera, unclassified *Lachnospiraceae* significantly decreased in Guatemala and unclassified *Ruminococcaceae* significantly decreased in Guatemala and DRC. The change in the overall community structure at the genus level was associated with a decrease in the abundances of certain genera with low heterogeneity among the four sites. Intervention arms were not significantly associated with inflammatory biomarkers at 12 or 34 weeks. AGP significantly decreased from 12 to 34 weeks of pregnancy, whereas CRP, MPO, and calprotectin did not significantly change over time. None of these biomarkers were significantly associated with the gut microbiota diversity.

**Conclusion:**

The longitudinal reduction of individual genera (both commensals and potential pathogens) and alpha-diversity among all sites were consistent and suggested that the effect of pregnancy on the maternal microbiota overrides other influencing factors, such as nutrition intervention, geographical location, diet, race, and other demographical variables.

## Introduction

The gut microbiome plays a critical role in human health and disease, including growth, body weight regulation, and immune function (Turnbaugh et al., 2006, 2009), especially during critical developmental phases. Pregnancy represents a time of altered metabolism, insulin sensitivity, immune response, and possibly the gut microbiota. Despite recent efforts and progress in elucidating the role of gut microbiota in human health and disease, there is a lack of consensus on changes in the gut microbiota during pregnancy. More importantly, whether this change is connected to metabolic health indicators, like inflammatory biomarkers, is still unclear. A limited number of studies have reported gut microbiota changes during pregnancy. One study in Finland found an increase in the microbial counts by qPCR from the first to third trimester (Collado et al., 2008). A landmark study using the same cohort (Koren et al., 2012) reported that while the gut microbiota in the first trimester was comparable to pre-pregnancy microbiota, there was a reduced diversity and increased abundance of pro-inflammatory strains, such as *Proteobacteria*, in the third trimester. Of note, participants in this study received probiotics and diet counseling (Koren et al., 2012). Several studies were published after this, producing inconsistent findings. Two studies on US women showed that gut microbiota remained relatively stable throughout pregnancy (DiGiulio et al., 2015; Dunlop et al., 2019). One Italian cohort showed an increase in diversity when comparing the second and third trimesters in women with gestational diabetes (Ferrocino et al., 2018).

Overall, current literature is limited and inconclusive, with different gut microbiota diversity and compositions. The discrepancies observed among these studies could partially be attributed to different analytical approaches, potential confounding variables (health status, diet, and probiotic use), and time of sample collection. These discrepancies warrant additional comprehensive and systematic assessments of gut microbiota changes during pregnancy, particularly in low-resource settings where data are lacking and health status, environment, and diet may differ from high-resource settings, and in relation to other health indicators that are reported to change over pregnancy, such as inflammation.

The Women First (WF) Preconception Maternal Nutrition Trial recruited women of reproductive age living in resource-limited environments [Democratic Republic of the Congo (DRC), India, Guatemala, and Pakistan] to participate in a preconception lipid-based nutrient supplementation trial (Hambidge et al., 2014). As part of the WF trial, biospecimens for the gut microbiota and systemic and intestinal inflammation were collected. Our previous report demonstrated distinctive dietary intakes and gut microbiota between non-pregnant WF participants in DRC and India prior to conception and initiation of the intervention (Tang et al., 2019). This report aims to determine the effect of pregnancy on the gut microbiota diversity and composition and inflammatory biomarkers in WF participants. We hypothesized that gut microbiota diversity and composition change over the course of pregnancy from the first to third trimester and would differ by intervention arm. Since pregnancy is known to cause changes in inflammation, we also examined potential associations between changes in gut microbiota and changes in systemic and intestinal inflammatory biomarkers during pregnancy.

## Methods

### Study Design

The WF trial aimed to determine whether a preconception nutrition supplement promotes better length and other anthropometric outcomes at birth (Hambidge et al., 2019). Women of child-bearing age were recruited from rural and semi-rural regions of DRC (Ubangi Province), Guatemala (Chimaltenango), India (Belagavi, Karnataka), and Pakistan (Thatta, Sindh Province). Enrolled participants were randomized to one of the following three arms: (1) started the daily supplement ≥3 months before conception and continued through delivery (Arm 1); (2) started the same intervention of daily supplement late in the first trimester until delivery (Arm 2); or (3) received no nutrition supplements besides those self-administered or prescribed through local health services (Arm 3). The supplement was a small-quantity lipid-based micronutrient supplement (SQ-LNS) containing ∼20 micronutrients and a favorable mix of PUFAs (Hambidge et al., 2019). Findings presented in the current report include the gut microbiota data of women who were pregnant within 24 months of enrollment. Stool and blood samples were collected from the participants at the end of the first and during the third trimesters. Participants in Arms 1 and 2 provided samples at both time points, while participants in Arm 3 provided samples at 34 weeks only. For the first trimester time point, specimens were collected from participants in Arm 2 prior to initiating the intervention supplement, and this group thus served as a control group at 12 weeks. In India, the sampling site did not collect samples from participants in Arm 3 at third trimester. At the DRC site, the blood sample was not collected due to a lack of cold chain facilities. Information on participants’ demographics, home environment, and family health history was also collected at enrollment. The WF trial was registered at clinicaltrials.gov (NCT01883193).

### Participants

Women of child-bearing age were recruited from the four sites based on the following inclusion criteria: age 16–35 years; parity 0–5, with site-specific strategies to include nulliparous participants; no current or planned contraceptive use; and expectation to conceive during the following 18 months. Informed consent was obtained from all participants. All participants who entered the pregnancy phase of the study consented to provide blood and fecal biospecimens. Hence, the women in the current study were primarily those who were the first to conceive.

### Blood Sample Collection

Blood samples were collected at 12 weeks (Arms 1 and 2) and at approximately 34 weeks (Arms 1, 2, and 3) of gestation. Specimens were collected using Sarstedt blood collection Safety-Multifly^®^ needles and serum S-Monovette^®^ blood collection tubes (Newton, NC, United States). The blood clotted for 30 min, was centrifuged, and then transferred to cryovials (Sarstedt). Serum was stored in –80°C freezers located at each site until shipment on dry ice to the University of Colorado Pediatric Nutrition Laboratory for analysis. Upon receipt, all samples were stored at –80°C until aliquoted and analyzed.

### Fecal Sample Collection

Two stool samples were collected from each participant during pregnancy. A pre-labeled fecal bag, Ziploc bag, a black cryogenic pen, and a Styrofoam storage box containing ice or ice packs were provided to each participant. The stool was collected into the fecal bag using a sterile scoop and then placed into a second Ziploc bag by participants. The bag was then placed into the Styrofoam storage container until picked up by the research team on the day the stool was passed. When receiving samples, the research team labeled the sample date and time of stool passage. The research team scooped about a teaspoon of stool and transferred the sample to a sterile stool storage tube with 3 mL RNAlater™, ensuring that the specimen was coated with RNAlater™ and that the label was complete. The stool samples were then frozen at –20°C or at a colder temperature. Samples were shipped to the University of Colorado on ice packs or at ambient temperature. Upon receipt, all samples were stored at –80°C until aliquoted and analyzed. Some samples were initially stored at –20°C but were transferred to permanent storage at –80°C. An additional fecal sample was collected without RNAlater™ for inflammatory biomarker analysis; this sample was frozen at –80°C, shipped on dry ice, and stored at –80°C at UCD until analysis.

### 16S rRNA Gene Sequencing

Bacterial profiles were determined by broad-range amplification and sequence analysis of the 16S rRNA gene following our previously described methods (Hara et al., 2012; Markle et al., 2013; Tang et al., 2017, 2019). In brief, amplicons were generated using primers that target approximately 450 base pairs of the V3–V4 variable region of the 16S rRNA gene (primers 338F: 5′ ACTCCTACGGGAGGCAGCAG and 806R: 5′ GGACTACHVGGGTWTCTAAT). PCR products were normalized using a SequalPrep™ kit (Invitrogen, Carlsbad, CA, United States), pooled, lyophilized, purified, and concentrated using a DNA Clean and Concentrator Kit (Zymo, Irvine, CA, United States). Pooled amplicons were quantified using Qubit Fluorometer 2.0 (Invitrogen, Carlsbad, CA, United States). Illumina paired-end sequencing was performed on the Miseq platform with versions v2.4 of the Miseq Control Software and MiSeq Reporter, using a 600 cycle version 3 reagent kit.

Illumina Miseq paired-end reads were aligned to human reference genome hg19 with bowtie2, and matching sequences were discarded (Homo Sapiens, 2009; Langmead and Salzberg, 2012). As previously described, the remaining non-human paired-end sequences were sorted by sample *via* barcodes in the paired reads with a python script (Tang et al., 2017, 2019). The sorted paired reads were assembled using phrap (Ewing et al., 1998; Ewing and Green, 1998). Pairs that did not assemble were discarded. Assembled sequence ends were trimmed over a moving window of five nucleotides until the average quality was met or exceeded 20. Trimmed sequences with more than one ambiguity or shorter than 350 nt were discarded. Potential chimeras identified with Uchime (usearch6.0.203_i86linux32) (Edgar et al., 2011) using the Schloss (Schloss and Westcott, 2011). Silva reference sequences were removed from the subsequent analyses. Assembled sequences were aligned and classified with SINA (1.3.0-r23838) (Pruesse et al., 2012) using the 418,497 bacterial sequences in Silva 115NR99 (Quast et al., 2013) as reference configured to yield the Silva taxonomy. Operational taxonomic units (OTUs) were produced by clustering sequences with identical taxonomic assignments. The average Good’s index of each sequence library was 99.7%, and the minimum Good’s index was 99.0%, indicating that the most biodiversity was captured in each library (participant) and the depth of sequencing was sufficient to represent the biodiversity in the specimens. A minimum read count of 3,700 was used to further filter libraries. The median number of read counts across all the samples was 96,064 with a minimum of 3,983, a maximum of 406,259, and an IQR of 82,504. The software package Explicet (v2.10.5) (Robertson et al., 2013) was used for display, microbial diversity analysis, and figure generation of results. The 16S rRNA gene sequence data and associated metadata have been deposited in the NCBI Sequence Read Archive under Bioproject ID PRJNA553183.

### Inflammatory Biomarker Analyses

Inflammatory biomarkers in the blood (AGP and CRP) and feces (MPO and calprotectin) were analyzed. C-reactive protein (CRP) was measured in serum samples from Guatemala and Pakistan using a sandwich immunoassay with electrochemiluminescent detection (Meso Scale Discovery, Gaithersburg, MD, United States, catalog #K151STD). The limit of detection was 0.034 mg/L, and within-day and between-day precisions were 5 and 6.4%, respectively. Samples from India were analyzed by immunoturbimetry (Siemens Dimension, Malvern, PA, United States) at the KLE Hospital Clinical Lab (Belagavi, Karnataka, India). The limit of detection was 0.6 mg/L, with a within-day precision of 1.1% and the between-day precision of 1.4%. Serum α-1-acid glycoprotein (AGP) was measured using a sandwich enzyme-linked immunoassay (R&D Systems, Bio-Techne Corporation, Minneapolis, MN, United States, catalog #DAGP00). For samples from Guatemala and Pakistan, the limit of detection was 0.001 μg/mL, and the within-day and between-day precisions were 4.8 and 14.2%, respectively. The limit of detection of the method used in India was 0.002 μg/mL, while the within-day sensitivity was 7.5% and the between-day sensitivity was 10.7%. To minimize potential batch effects, samples from Guatemala and Pakistan were balanced across runs by randomizing within the site, arm, and longitudinal time points in Excel. Samples from India were not randomized but instead run chronologically by participant ID, starting with 12-week and then 34-week time points. The study design, however, randomized participant ID across arms, which minimized batch effects.

The levels of calprotectin and myeloperoxidase were measured in the fecal samples. Calprotectin was measured using a two-step sandwich enzyme-linked immunoassay (ALPCO, Salem, NH, United States, catalog #30-CALPHU-EO1) without changes in the protocol. The within-day and between-day precisions were 4.6 and 12%, respectively. The limit of detection was 1.8 ng/mL. Myeloperoxidase was measured using a sandwich immunoassay with electrochemiluminescent detection (Meso Scale Discovery, Gaithersburg, MD, United States, catalog # K151EE), with the limit of detection of the method being 0.03 ng/mL, within-day precision being 4.5%, and between-day precision being 9%. We used cold 1% v/v 100× protease inhibitors (Sigma-Aldrich, St. Louis, MO, United States, catalog #P2714) in PBS (Sigma-Aldrich, St. Louis, MO, United States, catalog #P3813) as the extraction buffer. For the detection of both calprotectin and MPO, 5 mL of extraction buffer was added to 100 mg of stool, and the sample was vortexed at 1,500 rpm for 30 min. Extracts were then centrifuged at 3,000 *g* for 10 min at 4°C, then aliquoted, snap-frozen, and stored at –80°C until analysis. The amount of time between extraction and analysis was minimized and consistent to minimize the confounding of stability issues (1 day for MPO). To minimize the potential batch effects, samples were balanced across runs by site, arm, and time point.

### Statistical Approach

The longitudinal microbiome data were available for a subset of mothers. The mean age and BMI of these mothers were compared within each site to the mean age and BMI of all mothers from the respective site. A difference of means *t*-test was used to determine if these baseline characteristics were different among the two groups. Likewise, the distributions of SES and maternal education were compared at the site level between the two groups using the Cochran–Armitage test.

Data presented here were analyzed cross-sectionally and longitudinally. Analyses were performed stratified by the site for both alpha- and beta-diversity, and individual OTUs and results were combined using meta-analysis as described below. Statistical analyses were completed at the genus level. Across the entire dataset, OTUs present in at least 5% of all samples and with a relative abundance greater than 0.001 in at least one sample were retained for analysis. Microbiome information for the control arm (Arm 3) was not collected at 12 weeks of gestation. As a result, analyses comparing mothers across arm status could not be conducted. Instead, an intervention arm in the form of supplemental status at each time point was used to investigate the relationship between nutritional supplements and the microbiome. In this way, mothers from Arm 2 served as a control group at 12 weeks of gestation allowing for meaningful statistical comparisons. To be clear on how supplement status was assigned, at 12 weeks, women in Arm 1 had supplement status equal to 1, and women in Arm 2 had supplement status equal to 0. At 34 weeks, women in Arms 1 and 2 had supplement status equal to 1, and women in Arm 3 had supplement status equal to 0. When possible, we tested the interaction between supplement status and time. There were no samples from women in Arm 3 for India, thus it was not possible to test for the interaction between supplement and time for this site at 34 weeks of gestation. Given the assumption of no interaction, we were able to test for association between the microbiome and supplement and between the microbiome and time. All models included standardized read depth and batch as covariates.

Generalized estimating equations (GEEs) were used to model the association between time and supplement status and the interaction between time and supplement and four measures of alpha-diversity (Sobs, Chao 1, Shannon H, and Shannon H/Hmax) when controlling for site-specific covariates and repeated measures over individuals. PERMANOVA using 1,000 permutations with adjustments for repeated measures was used to assess the association of time or supplement status with beta-diversity controlling for site-specific covariates using the Bray-Curtis metric.

Individual OTUs were regressed on time and supplement status controlling for site-specific covariates using a negative binomial generalized linear model. Significance testing was performed by using 1,000 bootstrap samples to generate the distribution for effect estimates and then testing the hypothesis that the mean was equal to zero. The code used for this analysis is available on GitHub and can be accessed through the URL in the resources section.

As there were too many possible covariates to include for stable models and to account for differences between countries, we selected site-specific covariates as follows. Ninety-eight putative covariates were considered. Each covariate was assessed separately for each site and timepoint by using PERMANOVA to model beta-diversity as the outcome and the putative covariate of interest as a predictor. Each model was also adjusted for batch, regional cluster, and supplement status by including these parameters in the model. For each site, if the association between the covariate and beta-diversity had a *p*-value below 0.1 for at least one time point, it was considered further. The pairwise Pearson correlation for these putative covariates was then estimated within each site to avoid collinearity issues in the final models. Only one covariate was retained from covariate pairs with a correlation greater than 0.7. Within each pair, the putative covariate associated with most sites was kept. If the putative covariates were associated in the same number of sites, we considered whether the highly correlated covariates represented similar characteristics and selected the covariate that was more general (e.g., overall SES was kept instead of a single SES factor) to make our final list of covariates. Table 1 provides a summary of all covariates used in the final multivariable models by the site.

**TABLE 1:** List of covariates used in all final models by the site.

	DRC	Guatemala	India	Pakistan
Batch	X	X	X	X
Read depth	X	X	X	X
Overall SES	X			
Improved water source	X		X	
Delivery date	X			
Total years of schooling (mother)	X			
Supplement compliance			X	
Cooking fuel				X

To control for multiple testing, *p*-values were adjusted within site and analysis type (alpha-diversity, beta-diversity, or individual OTU) using the Benjamini–Hochberg method to control the false discovery rate (FDR) at 0.05 (Benjamini and Hochberg, 1995). All statistical analyses were performed using R version 4.0.3.

Meta-analyses across the four sites were performed at the genus level for individual OTUs, as well as alpha- and beta-diversity measures. Random-effects meta-analysis was performed for time and supplement terms for each OTU. For the four alpha-diversity measures, a random-effects meta-analysis was performed with an interaction between time and supplement, as well as for time and supplement terms without the interaction term included in the model. India was not included in this meta-analysis due to all women being on the supplement at 34 weeks. All random-effects meta-analyses were performed using the *metagen* function from the meta R package (Balduzzi et al., 2019). Meta-analysis for beta-diversity was performed using Fisher’s combination of *p*-values for the interaction term, as well as time and supplement with the interaction term removed. Forest plots were created for all OTUs and alpha-diversity meta-analyses using the *forest* function from the meta R package.

The differences in the relative abundance of the four most abundant phyla with respect to site location (the DRC, Guatemala, India, and Pakistan) and time (12 weeks of gestation compared to 34 weeks of gestation) are analyzed on the complete dataset (all site locations combined). All models at this taxonomic level are conditioned on the batch in which samples were analyzed, but no other covariates are used for adjustments. Two linear mixed-effects models are constructed for each phylum: one to fit the relationship of relative abundance with the interaction of time and site, the other to fit the relationship of relative abundance with time and site without considering the interaction of the two. ANOVA on the mixed-effects models is used to assess the strength of the association between the relative abundance and the factor of interest, starting with the interaction model and then moving to the additive model if the *p*-value for interaction is not below a threshold of 0.05. If the interaction has a strong association with relative abundance, *t*-tests for pairwise comparisons of the factor levels are performed and *p*-values are adjusted using Tukey’s adjustment. If the interaction was not strongly associated but one, or both, of the additive factors are strongly associated (i.e., *P* < 0.05) with the relative abundance, then the pairwise comparisons are performed using the additive model.

## Results

### Participants and Biospecimens

The recruitment of participants took place between December 2013 and October 2014. Out of the 3,251 participants who entered phase 2 (pregnancy) of the WF trial, stool samples were collected from 640 participants. These 640 participants were those who consented to biospecimen collection and were representative of the WF cohort. Table 2 summarizes the number of stool samples that were collected from the WF participants at 12 and 34 weeks of pregnancy. At 12 weeks, stool samples were collected from women in Arms 1 and 2 (prior to initiation of supplement). At 34 weeks, samples were collected from all three arms at all sites except for India, where samples were collected from Arms 1 and 2 only. Participants’ baseline age, BMI, education, and SES indicators are summarized in Table 3. Overall, the subcohort of WF participants in which gut microbiota was profiled was representative of the parent cohort (Hambidge et al., 2019). Comparing the included (women with microbiota data) vs. excluded (women without microbiota data), three variables at two sites were significantly different at a nominal 0.05 level: BMI in India (19.3 included vs. 20.2 excluded, *P* = 0.01), SES indicator in Pakistan (median 3 for included vs. median 2 for excluded, *P* = 0.01), and maternal education in India (median 3 for included vs. median 2 for excluded, *P* = 0.002).

**TABLE 2:** Number of microbiota samples analyzed by site, gestational time points, and supplement arms.

	First trimester (12 weeks)		Third trimester (34 weeks)			Total number of participants^a^
Arms	1	2	1	2	3	
DRC^b^	46	45	48	40	54	157
Guatemala	94	98	85	95	76	102
India	49	48	48	46	0	276
Pakistan	35	28	36	27	34	105
Total samples	224	219	217	208	164	640

*^a^Total number of participants that completed both first and third trimester sample collections.*

*^b^DRC, the Democratic Republic of the Congo.*

**TABLE 3:** Participants’ baseline characteristics by site.

	DRC^#^	India	Guatemala	Pakistan
Age, y*	24 ± 5 (0.58)	22 ± 3 (0.40)	24 ± 4 (0.24)	22 ± 4 (0.54)
BMI (kg/m^2^)*	20 ± 2 (0.64)	19± 3 (0.01)	25± 4 (0.49)	20 ± 3 (0.63)
Maternal education (*P*-value)	(0.84)	(0.002)	(0.69)	(0.81)
No school (n)	35	1	17	90
Primary (n)	92	11	188	10
Secondary or more (n)	27	90	71	5
Tally of indicators of higher SES (*P*-value)	(0.38)	(0.79)	(0.69)	(0.01)
None (n)	76	0	0	2
1–2 (n)	76	13	30	36
3–4 (n)	2	63	161	39
5–6 (n)	0	26	85	28
GWG (kg)^&^	4.7 (1.2)	6.2 (0.7)	7.8 (0.9)	6.5 (1.5)

*^#^DRC, the Democratic Republic of the Congo; SES, Socio-economic status.*

*Mean ± SD (P-value). P-values were for the comparisons of women with microbiota samples vs. all women from that site.

*^&^Gestational weight gain from first to third trimester.*

### Diversity of Gut Microbiota

**FIGURE 1:**
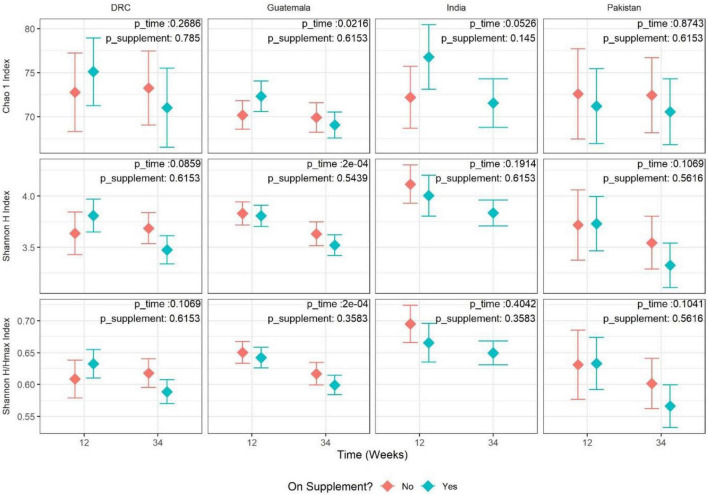
Adjusted mean estimate for alpha-diversity values by site and time (12 vs. 34 weeks). Each panel represents one pairing of site and alpha-diversity measure [eveness, richness, and Shannon diversity (richness and eveness)]. Each value is the adjusted mean estimate from the GEE model (diamond point) and 95% CI (error bars) for the respective measure of alpha-diversity split by time point and supplement status. Supplement status “Yes” indicates Arm 1 at 12 weeks and Arms 1+2 at 34 weeks. Supplement status “No” indicates Arm 2 at 12 weeks and Arm 3 at 34 weeks. The corresponding *p*-values for tests of time and supplement status are overlayed in the top-right corner of each panel.

Alpha-diversity was evaluated in terms of both richness (Chao1, the number of OTUs predicted based on observed singletons and doubletons), evenness (Shannon H/Hmax), and overall diversity of richness and evenness combined (Shannon H diversity index). Overall, there was insufficient evidence to conclude women taking the nutritional supplement had a different trend in alpha-diversity from 12 to 34 weeks than the control group (meta-analysis FDR-adjusted *p*-values of 0.289, 0.688, and 0.603 for richness, evenness, and overall diversity, respectively). There was evidence of a consistent lower overall diversity (Shannon H diversity index) in women taking the nutritional supplement compared to women not taking the supplement (meta-analysis estimate of –0.08 and meta-analysis FDR-adjusted *p*-value of 0.0276). Additionally, there was evidence for a consistent decrease in the Shannon H diversity from 12 to 34 weeks of gestation (meta-analysis estimate of –0.22 and meta-analysis FDR-adjusted *p*-value of 0.0197). In the stratified analysis of the Guatemalan cohort, there was strong evidence to conclude that richness, evenness, and overall diversity decreased from 12 to 34 weeks of gestation (richness estimate = –2.13, *P* = 0.0216; evenness estimate = –0.04, *P* = 0.0002; overall estimate = –0.25, *P* = 0.0002) (Figure 1). To summarize, women on supplements tended to have lower diversity (Shannon H) than women who were not on the supplement; from 12 to 34 weeks, gut microbiota diversity (Shannon H) tended to decrease. The strength of the relationship, however, was not always consistent across the four sites.

**FIGURE 2:**
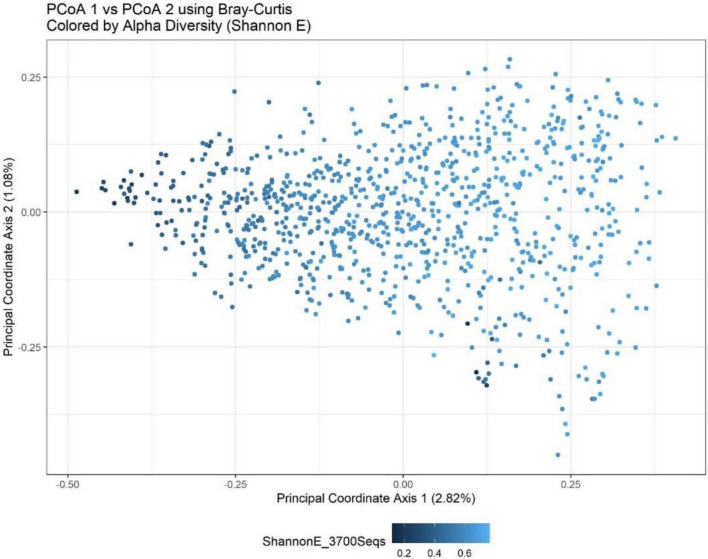
Bray-Curtis PCoA plot of all sites and timepoints colored by Shannon H index. PCoA1 shows a separation of observations based upon a measure of eveness. The percentage of variance explained by each axis is presented in parentheses within the figure legends. Together, the first two components explain just under 4% of the variation in the Bray-Curtis metric.

Beta-diversity values between observations from the same time point (12 weeks of gestation compared to 34 weeks of gestation) were more similar than the values between the time points in all four sites [DRC (*P* = 0.0009), Pakistan (*P* = 0.0009), Guatemala (*P* = 0.0009), and India (*P* = 0.034)]. Additionally, beta-diversity was seen to have a significant association with the supplement status in DRC (*P* = 0.003) and Pakistan (0.0009). However, there was insufficient evidence to conclude that the interaction between time and supplement status is associated with beta-diversity in any of the sites. These results are better understood through a PCoA plot (Figure 2) that visualizes the first two principal coordinate axes that are constructed using observations from all four sites and then colored by species evenness (Shannon E). PCoAs 1 and 2 contributed to roughly 4% of the overall variability, which is a relatively low percentage of total variability and indicates it will be difficult to project relationships within the data to a two-dimensional figure. However, it appears that PCoA axis 1 separates observations by alpha-diversity, with smaller values on the left and larger values on the right. Because evenness was associated with supplement and time, it is reasonable to conclude that the beta-diversity results and the alpha-diversity results are being driven by the same underlying taxa.

### Gut Microbiota Community Structure

The top four most abundant phyla, accounting for >95% of sequences, were Firmicutes, Bacteroidetes, Proteobacteria, and Actinobacteria for all four sites at both time points in pregnancy (Table 4). Relative abundances at the genus level are summarized in Supplementary Figure 1. Among the most abundant genera, there were no statistically significant relationships between taxa count and supplement status or time point after adjusting for multiple testing, which was consistent in all four sites. In the stratified analyses, unclassified *Ruminococcaceae* showed a significant decrease in abundance during pregnancy in DRC (estimate = –0.442, FDR *P* = 0.0003) and Guatemala (estimate = –0.389, FDR *P* = 0.00002). *Prevotella* and unclassified *Lachnospiraceae* were also significantly associated with time in the Guatemala cohort: *Prevotella* increased from 12 to 34 weeks (estimate = 0.326, FDR *P* = 0.000005) and unclassified *Lachnospiraceae* decreased from 12 to 34 weeks (estimate = –0.236, FDR *P* = 0.0165). Among the top 10 most abundant genera, there was not enough evidence to conclude an association between abundance and supplement status in the stratified analyses. A broader, genus-level analysis showed numerous genera that significantly (FDR *P* < 0.05) changed in relative abundance from the first to third trimester (Supplementary Table 1) or changed by supplement status (Supplementary Table 2) in at least one site. A meta-analysis of all sites combined was conducted at the broader genus level, and the results of significant changes are summarized in Table 5. These results show a decrease in the abundances of certain genera with consistent significant effects observed among the four sites.

**TABLE 4:** The top four most abundant phyla at the first and third trimesters by the site^a,d^.

Phyla	DRC^b^		Guat^b^		India		Pakistan	
	First^c^	Third^c^	First	Third	First	Third	First	Third
Firmicutes^e^	56% (17.9%)	55% (17.9%)	56% (13.8%)	49% (15.2%)	53% (14.4%)	48% (14.7%)	54% (17.4%)	47% (16.4%)
Bacteroidetes^f,g^	27% (17.1%)	31% (18.3%)	32% (14.3%)	40% (16.6%)	28% (14.8%)	31% (16.2%)	29% (18.0%)	38% (20.0%)
Actinobacteria^g^	2% (10.7%)	2% (7.6%)	1% (2.2%)	2% (3.9%)	6% (7.6%)	8% (12.6%)	7% (9.1%)	5% (10.2%)
Proteobacteria^e^	9% (11.6%)	9% (10.4%)	6% (6.9%)	5% (5.6%)	9% (8.6%)	10% (9.6%)	7% (10.0%)	7% (7.1%)

*^a^Values are average relative abundance (standard error).*

*^b^DRC, the Democratic Republic of the Congo; Guat, Guatemala.*

*^c^First, first trimester (12 weeks); third, third trimester (34 weeks).*

*^d^ANOVA tests for significant factor levels in Supplementary Table 3.*

*^e^Interaction between site and time is statistically significant; comparisons in Supplementary Tables 4, 5.*

*^f^Trimester is statistically significant; comparisons in Supplementary Table 6.*

*^g^Site is statistically significant; comparisons in Supplementary Tables 7, 8.*

**TABLE 5:** Meta-analysis of all sites combined for the genera remained significant.

Genera	Average relative abundances	Meta-analysis effect	Standard error	FDR *P*-value
Christensenellaceae	0.933%	–0.7	0.02	0.0064
VadinHA64	0.012%	–1.39	0.04	0.0116
Enterococcaceae	0.001%	–1.98	0.07	0.015

### Inflammation During Pregnancy

The inflammatory biomarkers in the blood (AGP and CRP) and stool [myeloperoxidase (MPO) and calprotectin] were analyzed in a subgroup of participants from whom stool samples were collected. Inflammatory markers were not measured in the DRC due to limited access to the cold chain facility. Table 6 presents these inflammatory biomarkers by site and stage of pregnancy. Supplement status did not significantly affect the inflammatory biomarkers. AGP significantly decreased from 12 to 34 weeks of pregnancy and other inflammatory biomarkers did not significantly differ over pregnancy. These biomarkers were not significantly associated with the alpha-diversity values of gut microbiota.

**TABLE 6:** AGP, CRP, MPO, and calprotectin in first and third trimester by the site^a^.

Site (sample size)	First trimester (12 weeks)			Third trimester (34 weeks)		
	Pakistan (59)	India (97)	Guatemala (143)	Pakistan (86)	India (94)	Guatemala (200)
AGP^b^ (μg/mL)	871 ± 305	990 ± 371	915 ± 362	565 ± 198	597 ± 243	643 ± 220
CRP (mg/L)	7.2 ± 24	6.2 ± 13	9.8 ± 11	5.0 ± 6	5.6 ± 8	11.1 ± 16
MPO (ng/mL)	1,020 ± 2,607	1,112 ± 1,608	2,615 ± 4,481	1,306 ± 5,073	754 ± 1,789	2,091 ± 3,838
Calprotectin (μg/g)	27 ± 57	45 ± 57	81 ± 116	34 ± 59	40 ± 78	60 ± 67

*^a^Mean ± SD. AGP, alpha (1)-acid glycoprotein; CRP, C-reactive protein; MPO, myeloperoxidase.*

*^b^Significant change over time (P = 0.0003).*

## Discussion

The extent to which gut microbiota changes during pregnancy remains an open question, and most existing studies have been conducted in high-resource settings. Our study included low-resource settings in four countries with distinctive cultural and dietary backgrounds (Tang et al., 2019). For example, the communities within the DRC site predominantly included rural participants with a very low SES status compared to the other sites. In addition, most participants from India followed a lacto-vegetarian diet, whereas participants in other countries were more likely to consume some animal-sourced foods. We previously reported distinct gut microbiota diversity and composition between WF participants in DRC and India at enrollment before conception and prior to nutritional intervention. At the baseline/enrollment visit, differences in microbiota were associated with a complex set of environmental factors, dietary patterns, nutritional status, and race/ethnicity (Tang et al., 2019). Dietary intakes reported in the previous analysis (Tang et al., 2019) were assessed during the first trimester of the WF participants and showed different food groups and nutrient intakes between women from the DRC and India that were associated with differences in gut microbiota. A subsequent report of all four sites also showed that dietary patterns (exclusive of LNS supplements) varied widely among sites but were uniformly lacking in multiple essential nutrients, such as folate, vitamin B12, and choline (Lander et al., 2019). In our current analysis of gut microbiota changes during pregnancy, despite the differences in diet and other demographical factors, a conserved shift in both diversity and community compositions was observed among the four sites from the first to third trimester. These changes were mostly consistent among the four sites. These findings suggest that there may be consistent overall effects of pregnancy on the gut microbiota that persist even in the context of different cultural and dietary backgrounds or supplementation with LNS.

Alpha-diversity values, as assessed by the Shannon H index, showed a statistically significant decline from the first to third trimester across all the study sites. Earlier research has been inconsistent regarding alpha-diversity changes during pregnancy. A Finnish cohort showed a significant reduction in richness from the first to the third trimester (Koren et al., 2012). However, most of the subsequent studies did not observe the same reduction in microbial richness. Three US (DiGiulio et al., 2015; Dunlop et al., 2019; Ruebel et al., 2021) and one Chinese (Yang et al., 2020) cohort showed that microbial diversity remained stable during pregnancy from the first to the third trimester. A concept proposed on the basis of the Finnish cohort findings is that the decreased gut microbiota diversity over the period of pregnancy is associated with increased fat storage and insulin resistance, which is considered beneficial for fetal growth and subsequent lactation (Koren et al., 2012). In other words, the decreased alpha-diversity during pregnancy may be a physiological adaptation. The reasons for these discrepant observations among cohorts are not clear, but these studies did differ in sample size, geographical location, and maternal diets. The significant meta-analysis of alpha-diversity in the present study was driven primarily by the site in Guatemala as shown in the stratified analysis (Figure 1), although the other three sites followed the same trend. This could be due to the Guatemala cohort having the largest sample size resulting in a smaller standard error of the estimate. The women in the Guatemala site also had significantly higher BMI and the lowest risk of being underweight compared to the other three sites. It is probable that this physiological adaptation of a decrease in alpha-diversity only occurs in the context of adequate energy intake during pregnancy (e.g., Guatemala vs. the other three sites). The dietary assessment did show that women from the Guatemalan site had a greater energy intake (Lander et al., 2019). Two published studies examined the gut microbiota change from the second to the third trimester. In Tanzanian women, gut microbiota remained stable from the second to third trimester and was not affected by the consumption of probiotics in the form of yogurt (Bisanz et al., 2015). In contrast, Italian women with gestational diabetes (GDM) had an increase in alpha-diversity from the second to third trimester (Ferrocino et al., 2018). Thus, gut microbiota during pregnancy may change differently with different maternal metabolic health statuses.

It is also important to consider potentially masked effects due to outliers within the data studied in this manuscript. All reported results come from statistical models fit using site-stratified observations after performing microbiome quality checks. For alpha-diversity, sensitivity analysis of the model was performed by removing outliers defined as a residual value below *Q*_1_−3*IQR or above*Q*_3_ + 3*IQR where *Q*_1_ is the 25th percentile of the residuals, *Q*_3_ is the 75th percentile of the residuals, and *IQR* is the inter-quartile range (*Q*_3_−*Q*_1_) of the residuals. After removing outliers, results were consistent in all sites except for the DRC where there was evidence for changes over pregnancy in several measures when outliers were removed but not when outliers were included: Chao 1 (full = –2.09 and FDR-adjusted *p* = 0.218, outliers removed = –3.55 and FDR-adjusted *p* = 0.0126), Shannon H/Hmax (full = –0.021 and FDR-adjusted *p* = 0.0712, outliers removed = –0.024 and FDR-adjusted *p* = 0.0248). This finding suggests that outliers in the DRC cohort may be masking the association between alpha-diversity and weeks of pregnancy (gestation). The decline in alpha-diversity between the first and third trimesters was consistent with the overall decrease in the abundance of several genera (Table 1).

Consistent with our findings at the sites in DRC and India before pregnancy (Tang et al., 2019), *Prevotella* remained the most abundant genus among all sites during pregnancy, reflecting the semi-rural setting and diets, and increased in Guatemala over the course of pregnancy. Out of the top 10 most abundant genera, unclassified *Lachnospiraceae* and *Ruminococcaceae* were significantly diminished in relative abundance from the first to the third trimester in Guatemala and DRC. These findings are consistent with the Finnish cohort, which also showed a decline in unclassified *Lachnospiraceae* and *Ruminococcaceae* (Koren et al., 2012), and the Chinese cohort showed a decline in unclassified *Lachnospiraceae* (Yang et al., 2020). Many species of *Ruminococcaceae* and *Lachnospiraceae* families degrade polysaccharides and produce short-chain fatty acids (SCFAs), which are a key source of nutrients to the host (Magne et al., 2006) and can directly regulate fat accumulation and energy expenditure of the host (Kimura et al., 2014). The population of *Ruminococcaceae* was reduced during the first trimester in women with hyperglycemia compared with women with normal glucose control in 50 Chinese women (Gao et al., 2020). In contrast, the abundance of the *Ruminococcaceae* family was associated with higher odds of a positive GDM diagnosis in 75 Finnish women (Mokkala et al., 2017). Nevertheless, the *Ruminococcaceae* family may be important in maintaining a healthy metabolic profile during pregnancy. In our meta-analysis, unclassified *Ruminococcaceae* had a moderately strong association with time based on the FDR *P*-value (*P* = 0.07). Further investigation is needed to identify the unclassified genera in the *Ruminococcaceae* and *Lachnospiraceae* families that decreased during pregnancy.

Another genus, *Blautia*, a probiotic and SCFA producer, also decreased in Guatemalan participants during pregnancy. In the Italian GDM cohort, both *Blautia* and *Lachnospiraceae* increased from the second to third third trimester (Ferrocino et al., 2018). In the Chinese cohort (Yang et al., 2020), *Blautia* was also enriched in women with GDM vs. without GDM. Two recent studies (Ye et al., 2019; Soderborg et al., 2020) also showed that *Blautia* was higher in women with GDM compared with non-GDM women, although the literature is not consistent. These changes, together with those observed in *Ruminococcaceae* and *Lachnospiraceae* in Guatemala, suggest that these putative SCFA-producing bacteria may be related to glycemic control during pregnancy, and a reduction in abundance might be beneficial for glucose regulation. In the WF participants, who on average had relatively low gestational weight gain and BMI (Bauserman et al., 2021), these genera decreased during pregnancy. Future research, for example, utilizing germ-free mice, is needed to explore the potential causal relation between these SCFA producers and glycemic control during pregnancy.

During late pregnancy, pro-inflammatory cytokines may increase (Kirwan et al., 2002) and insulin resistance may increase (Dahlgren, 2006). These metabolic changes are considered normal and help in maintaining a sufficient nutrient supply to the fetus. The Finnish cohort showed that a decreased gut microbiota richness was associated with low-grade inflammation during the third trimester (Koren et al., 2012). In the present study, we saw a decrease in AGP concentration among all sites. This is in contrast to previous findings and might be partially due to the very large variance observed in the inflammatory biomarkers (per timepoint per site) or an effect of hemodilution on pregnancy. CRP, on average, was elevated among all sites during pregnancy but did not change over time. Our values were similar to a previous study reporting CRP at 24 weeks of pregnancy in Denmark (Fink et al., 2019). The effects of maternal intestinal inflammation and its relationship to the microbiota during pregnancy, particularly in women in LMIC, warrant systematic investigations.

The strengths of this study include longitudinal assessment of gut microbiota during pregnancy (first and third trimester); inclusion of a relatively large sample size from four different countries across Africa, Asia, and Central America; and comprehensive analysis of covariates, including the LNS nutrition intervention of the primary trial. Limitations include lack of control arm for the site in India at 34 weeks of gestation; lack of blood from the DRC site; and only two time points of assessment.

In summary, the longitudinal reduction of individual genera (both commensals and potential pathogens) and alpha-diversity were consistent among all sites and suggests that the effect of pregnancy on the maternal microbiota overrides other influencing factors, such as geographical location, diet and nutrition supplement, race, and other demographical variables. Future research is needed to explore the associations of gut microbiota with other health indicators during pregnancy and in the offspring.

## Data Availability Statement

The datasets presented in this study can be found in online repositories. The names of the repository/repositories and accession number(s) can be found below: https://www.ncbi.nlm.nih.gov/, PRJNA553183.

## Ethics Statement

The studies involving human participants were reviewed and approved by the Colorado Multiple Institutional Review Board (COMIRB). Local ethics approval was obtained at all research sites. The patients/participants provided their written informed consent to participate in this study.

## Author Contributions

KMH and NK conceived the original idea and designed the initial protocol. AG, LF, AT, AL, JW, SG, MS, SA, and SS conducted the procedures. DF, DI, CR, and JK analyzed the stool samples and processed the microbiota data. NW, AH, DF, MT, and EM performed the statistical analysis. MT wrote the report with close collaboration with NK, DF, AH, JW, and NW, and with input from all authors. All authors read and approved the final version of the manuscript.

## Conflict of Interest

The authors declare that the research was conducted in the absence of any commercial or financial relationships that could be construed as a potential conflict of interest.

## Publisher’s Note

All claims expressed in this article are solely those of the authors and do not necessarily represent those of their affiliated organizations, or those of the publisher, the editors and the reviewers. Any product that may be evaluated in this article, or claim that may be made by its manufacturer, is not guaranteed or endorsed by the publisher.
