# Supplementary material for: Longitudinal Reduction in Diversity of Maternal Gut Microbiota During Pregnancy Is Observed in Multiple Low-Resource Settings: Results From the Women First Trial
Source: Front Microbiol. 2022 Aug 1;13:823757. doi: 10.3389/fmicb.2022.823757 (PMC9376441; doi:10.3389/fmicb.2022.823757)
Supplement: Supplementary file 1 [file Data_Sheet_1.docx]

**Supplementary materials**

**Supplementary Figure 1.** Relative abundances at the genus level by time and and by site. The top nine most abundant taxa (on average) are shown with the remaining taxa grouped into the ‘other’ classification. *Ruminococcaceae* and *Lachnospiraceae* represents unclassifed *Ruminococcaceae* and *Lachnospiraceae* at the genus level.

**
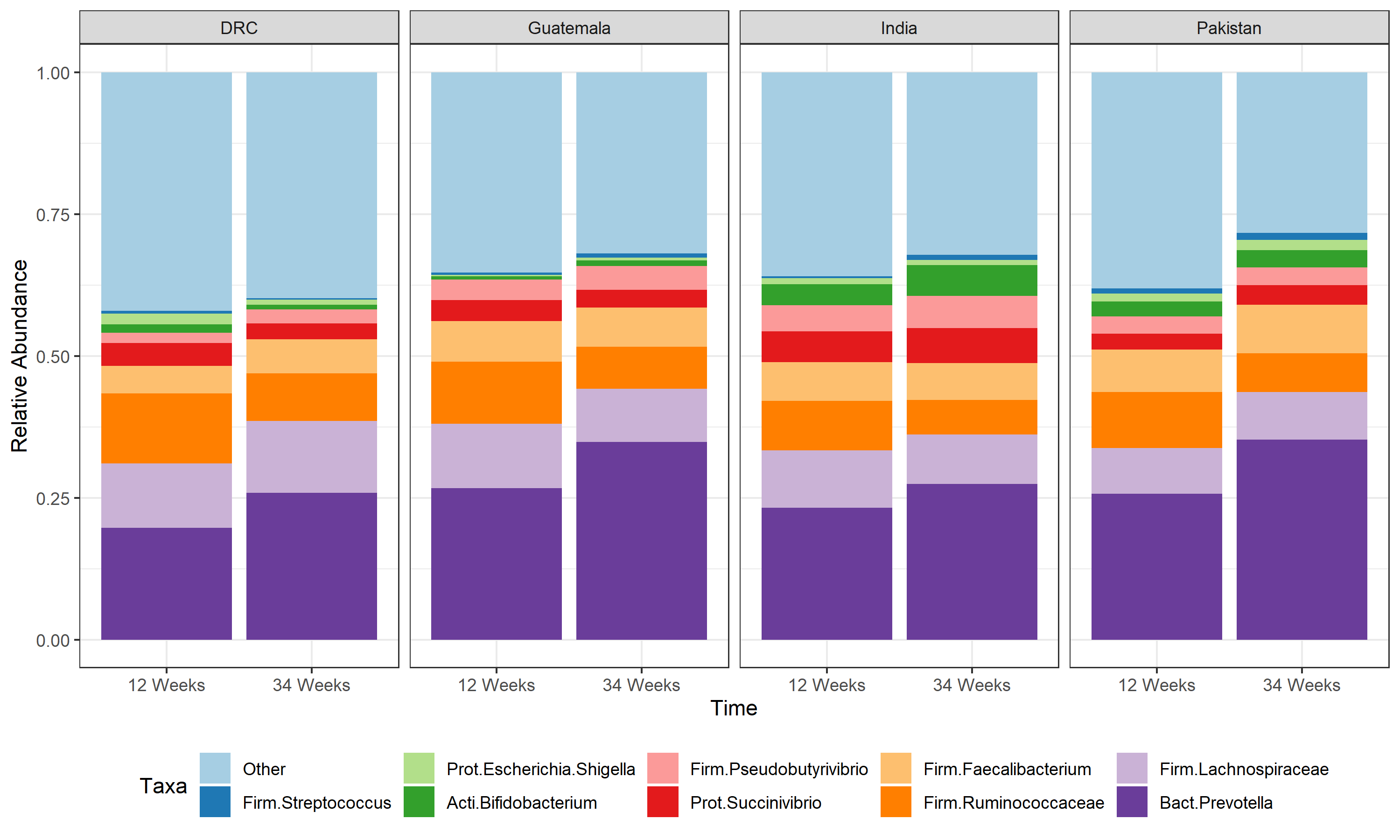
**

**DRC: Democratic Republic of the Congo**

**Supplementary Table 1**. Genera that had a significant change from 1^st^ to 3^rd^ trimester in at least one site^1^

| **Genera** | **DRC^2^** | **India** | **Guatemala** | **Pakistan** |
| --- | --- | --- | --- | --- |
| Actinomyces | ↔ [-0.79, 0.19] | ↓ [-1.07, -0.24] | ↔ [-0.27, 0.18] | ↔ [-1.84, -0.04] |
| Akkermansia | ↔ [-3.07, -0.10] | ↔ [-2.98, 0.71] | ↓ [-3.07, -0.97] | ↔ [-3.99, 5.40] |
| Alcaligenaceae | ↔ [-2.44, 1.44] | ↔ [-1.55, 27.08] | ↔ [-0.55, 2.29] | ↓ [-55.20, -0.91] |
| Alistipes | ↓ [-1.36, -0.51] | ↔ [-0.82, 0.77] | ↓ [-1.32, -0.51] | ↔ [-2.04, 0.91] |
| Anaerococcus | ↓ [-2.91, -0.74] | ↔ [-0.48, 2.90] | ↔ [-0.92, 1.69] | ↔ [-37.47, 8.73] |
| Anaerotruncus | ↓ [-1.40, -0.64] | ↔ [-1.13, -0.08] | ↓ [-0.84, -0.39] | ↔ [-1.23, 0.46] |
| Bacteria | ↔ [-0.48, 0.06] | ↔ [-0.51, 0.27] | ↓ [-0.88, -0.18] | ↔ [-1.19, 0.22] |
| Bilophila | ↔ [-1.05, -0.21] | ↔ [-0.78, 0.28] | ↓ [-1.13, -0.31] | ↔ [-3.25, 0.52] |
| Blautia | ↔ [-0.16, 0.38] | ↔ [-0.86, 0.05] | ↓ [-0.38, -0.11] | ↔ [-0.95, 0.23] |
| Butyricimonas | ↓ [-1.25, -0.34] | ↔ [-1.26, 0.21] | ↓ [-0.98, -0.21] | ↔ [-1.42, 1.14] |
| Butyrivibrio | ↔ [-0.99, -0.00] | ↓ [-2.25, -0.39] | ↔ [-0.61, 0.04] | ↔ [-2.17, 1.52] |
| Christensenellaceae | ↓ [-0.97, -0.45] | ↔ [-1.13, -0.13] | ↓ [-0.94, -0.48] | ↔ [-1.58, 0.14] |
| Clostridiales | ↔ [-0.21, 0.16] | ↔ [-0.66, -0.03] | ↔ [-0.38, -0.7] | ↓ [-1.07, -0.27] |
| Comamonas | ↔ [-1.72, 0.83] | ↓ [-4.06, -1.12] | ↔ [-1.64, 0.93] | ↔ [0.47, 8.06] |
| Elusimicrobium | ↓ [-2.16, -0.44] | ↔ [-1.41, 2.02] | ↔ [-1.16, 0.39] | ↔ [-5.30, 1.41] |
| Enterococcaceae | ↔ [-3.69, 0.14] | ↔ [-31.35, 21.46] | ↓ [-3.18, -0.92] | ↔ [-38.00, 4.36] |
| Family 8 Incertae Sedis | ↓ [-0.84, -0.30] | ↔ [-0.92, -0.10] | ↓ [-0.59, -0.23] | ↔ [-1.33, 0.24] |
| Haemophilus | ↔ [-0.79, 0.09] | ↓ [-1.69, -0.37] | ↔ [-0.29, 0.32] | ↔ [-1.42, 0.81] |
| Lachnospira | ↑ [0.43, 1.10] | ↔ [-0.46, 0.55] | ↔ [-0.14, 0.35] | ↔ [-1.46, 0.44] |
| Lachnospiraceae | ↔ [-0.19, 0.16] | ↔ [-0.42, 0.08] | ↓ [-0.37, -0.09] | ↔ [-0.65, 0.06] |
| Marvinbryantia | ↓ [-0.80, -0.21] | ↔ [-0.86, 0.03] | ↓ [-0.66, -0.27] | ↔ [-0.94, 0.26] |
| Mogibacterium | ↔ [-1.69, 0.35] | ↓ [-3.65, -0.70] | ↔ [-0.64, 0.28] | ↔ [-4.05, 4.07] |
| Odoribacter | ↔ [-1.19, 0.36] | ↔ [-0.62, 0.66] | ↓ [-1.25, -0.43] | ↔ [-3.52, 0.29] |
| Oribacterium | ↓ [-1.61, -0.61] | ↔ [-1.54, -0.20] | ↔ [-0.42, 0.22] | ↔ [-1.11, 0.22] |
| Oxalobacter | ↓ [-0.93, -0.26] | ↔ [-1.70, 0.08] | ↓ [-0.72, -0.20] | ↔ [-4.64, 0.98] |
| Parabacteroides | ↓ [-1.17, -0.29] | ↔ [-0.40, 0.71] | ↔ [-0.56, -0.02] | ↔ [-1.45, 0.99] |
| Prevotella | ↔ [0.00, 0.47] | ↔ [-0.29, 0.29] | ↑ [0.21, 0.44] | ↔ [-0.45, 0.61] |
| Prevotellaceae | ↔ [-0.05, 0.58] | ↔ [-1.43, 0.35] | ↑ [0.18, 0.60] | ↔ [-1.09, 0.25] |
| RF9 | ↓ [-1.04, -0.32] | ↔ [-1.46, -0.08] | ↓ [-0.90, -0.22] | ↔ [-2.13, 0.52] |
| Ruminococcaceae | ↓ [-0.62, -0.26] | ↔ [-0.89, -0.17] | ↓ [-0.55, -0.25] | ↔ [-1.18, 0.06] |
| Ruminococcus | ↔ [-0.17, 0.31] | ↔ [-0.79, 0.01] | ↓ [-0.56, -0.13] | ↔ [-1.97, -0.36] |
| S24_7 | ↓ [-0.89, -0.21] | ↔ [-0.50, 0.38] | ↔ [-0.71, -0.03] | ↔ [-1.55, 0.14] |
| Sarcina | ↔ [-0.73, 1.85] | ↔ [-0.59, 1.89] | ↑ [0.25, 1.19] | ↔ [-3.82, 1.21] |
| VadinBB60 | ↓ [-1.25, -0.46] | ↔ [-1.48, -0.23] | ↓ [-0.99, -0.48] | ↔ [-2.51, -0.13] |
| VadinHA64 | ↔ [-2.12, -0.66] | ↔ [-2.54, 0.10] | ↓ [-2.10, -0.64] | ↔ [-4.30, 1.80] |
| VC2_1 Bac22 | ↔ [-2.03, -0.27] | ↔ [-1.32, 1.62] | ↔ [-2.37, 0.37] | ↓ [-30.52, -0.24] |

^1^Values are 95% confidence intervals for estimate of association

^2^DRC: Democratic Republic of the Congo

**Supplementary Table 2**. Genera that had a significant change by for women on the nutritional supplement in at least one site^1^

| **Genera** | **DRC^2^** | **India** | **Guatemala** | **Pakistan** |
| --- | --- | --- | --- | --- |
| Comamonas | ↔ [-2.50, 0.32] | ↔ [0.46, 3.80] | ↔ [-1.03, 1.47] | ↓ [-3.64, -0.48] |
| Enterorhabdus | ↓ [-0.86, -0.18] | ↔ [-1.05, 1.78] | ↔ [-0.35, 0.32] | ↔ [-0.89, 0.09] |
| Odoribacter | ↔ [-0.76, 0.95] | ↓ [-1.49, -0.26] | ↔ [-0.59, 0.19] | ↔ [-1.60, 0.56] |
| Ruminococcus | ↓ [-0.66, -0.14] | ↔ [-0.44, 0.39] | ↔ [-0.20, 0.22] | ↔ [-0.99, 0.32] |
| Treponema | ↔ [-1.03, 2.12] | ↔ [-5.59, 8.13] | ↑ [0.37, 1.62] | ↔ [-1.04, 1.13] |

^1^Values are 95% confidence intervals for estimate of association

^2^DRC: Democratic Republic of the Congo

**Supplementary Table 3**. Analysis of Variance (ANOVA) results for Phylum models^1^

| **Phylum** | **Effect** | **F Statistic** | **P-value^2^** |
| --- | --- | --- | --- |
| Bacteroidetes | Trimester | 31.944 | < 0.0001 |
| Actinobacteria | Site | 12.367 | < 0.0001 |
| Bacteroidetes | Site | 6.095 | 0.0004 |
| Firmicutes | Interaction | 3.731 | 0.0112 |
| Proteobacteria | Interaction | 3.655 | 0.0125 |
| Actinobacteria | Trimester | 2.495 | 0.1148 |
| Bacteroidetes | Interaction | 1.882 | 0.1313 |
| Actinobacteria | Interaction | 1.666 | 0.1732 |

^1^Interaction of site and trimester models are created for all four phyla. Actinobacteria and Bacteroidetes also have individual trimester and site rows because the interaction term was not significant

^2^P-values are sorted from smallest to largest

**Supplementary Table 4**. Pairwise comparison tests for different combinations of site and trimester when considering the relative abundance of Firmicutes

| **Comparison^2^** | **Estimate** | **Tukey adjusted P-value^1^** |
| --- | --- | --- |
| Guatemala 12Weeks - Guatemala 34Weeks | 0.102 | < 0.0001 |
| DRC 34Weeks - India 34Weeks | 0.096 | 0.0352 |
| India 12Weeks - India 34Weeks | 0.068 | 0.0675 |
| DRC 34Weeks - Guatemala 34Weeks | 0.072 | 0.1489 |
| Pakistan 12Weeks - Pakistan 34Weeks | 0.099 | 0.2732 |
| Guatemala 12Weeks - India 12Weeks | 0.057 | 0.3578 |
| India 12Weeks - Pakistan 12Weeks | -0.091 | 0.5941 |
| India 34Weeks - Pakistan 34Weeks | -0.061 | 0.6128 |
| DRC 34Weeks - Pakistan 34Weeks | 0.035 | 0.8617 |
| DRC 12Weeks - India 12Weeks | 0.045 | 0.8999 |
| Guatemala 34Weeks - Pakistan 34Weeks | -0.037 | 0.9286 |
| Guatemala 34Weeks - India 34Weeks | 0.024 | 0.9619 |
| DRC 12Weeks - Pakistan 12Weeks | -0.046 | 0.9754 |
| DRC 12Weeks - DRC 34Weeks | 0.018 | 0.9892 |
| Guatemala 12Weeks - Pakistan 12Weeks | -0.034 | 0.9949 |
| DRC 12Weeks - Guatemala 12Weeks | -0.012 | 0.9999 |

^1^P-values are sorted from smallest to largest

^2^DRC: Democratic Republic of the Congo; 12Weeks: 1^st^ Trimester; 34Weeks: 3^rd^ Trimester

**Supplementary Table 5**. Pairwise comparison tests for different combinations of site and trimester when considering the relative abundance of Proteobacteria

| **Comparison^2^** | **Estimate** | **Tukey adjusted P-value^1^** |
| --- | --- | --- |
| Guatemala 34Weeks - India 34Weeks | -0.047 | 0.0022 |
| DRC 12Weeks - Pakistan 12Weeks | 0.090 | 0.0055 |
| DRC 34Weeks - Guatemala 34Weeks | 0.047 | 0.0301 |
| DRC 12Weeks - Guatemala 12Weeks | 0.051 | 0.0325 |
| Pakistan 12Weeks - Pakistan 34Weeks | -0.066 | 0.0451 |
| India 12Weeks - Pakistan 12Weeks | 0.073 | 0.1028 |
| Guatemala 12Weeks - India 12Weeks | -0.033 | 0.2702 |
| Guatemala 34Weeks - Pakistan 34Weeks | -0.030 | 0.6257 |
| Guatemala 12Weeks - Pakistan 12Weeks | 0.040 | 0.7022 |
| DRC 34Weeks - Pakistan 34Weeks | 0.017 | 0.9140 |
| India 12Weeks - India 34Weeks | -0.011 | 0.9774 |
| India 34Weeks - Pakistan 34Weeks | 0.017 | 0.9819 |
| DRC 12Weeks - India 12Weeks | 0.018 | 0.9826 |
| DRC 12Weeks - DRC 34Weeks | 0.007 | 0.9972 |
| Guatemala 12Weeks - Guatemala 34Weeks | 0.004 | 0.9997 |
| DRC 34Weeks - India 34Weeks | < 0.001 | > 0.9999 |

^1^P-values are sorted from smallest to largest

^2^DRC: Democratic Republic of the Congo; 12Weeks: 1^st^ Trimester; 34Weeks: 3^rd^ Trimester

**Supplementary Table 6**. Pairwise comparison test for difference in the relative abundance of Bacteroidetes between the first and third trimester of pregnancy

| **Comparison^2^** | **Estimate** | **Tukey adjusted P-value^1^** |
| --- | --- | --- |
| 12Weeks - 34Weeks | -0.065 | < 0.0001 |

^1^P-values are sorted from smallest to largest

^2^12Weeks: 1^st^ Trimester; 34Weeks: 3^rd^ Trimester

**Supplementary Table 7**. Pairwise comparison tests for differences in the relative abundance of Bacteroidetes between the four sites

| **Comparison^2^** | **Estimate** | **Tukey adjusted P-value^1^** |
| --- | --- | --- |
| DRC - Guatemala | -0.106 | 0.0003 |
| Guatemala - Pakistan | 0.089 | 0.0178 |
| DRC - India | -0.065 | 0.1173 |
| Guatemala - India | 0.041 | 0.1678 |
| India - Pakistan | 0.048 | 0.4899 |
| DRC - Pakistan | -0.018 | 0.8898 |

^1^P-values are sorted from smallest to largest

^2^DRC: Democratic Republic of the Congo

**Supplementary Table 8**. Pairwise comparison tests for differences in the relative abundance of Actinobacteria between the four sites

| **Comparison** | **Estimate** | **Tukey adjusted P-value^1^** |
| --- | --- | --- |
| Guatemala - India | -0.043 | < 0.0001 |
| Guatemala - Pakistan | -0.052 | 0.0004 |
| DRC - Pakistan | -0.028 | 0.0403 |
| DRC - Guatemala | 0.024 | 0.1349 |
| DRC - India | -0.019 | 0.4402 |
| India - Pakistan | -0.009 | 0.9213 |

^1^P-values are sorted from smallest to largest

^2^DRC: Democratic Republic of the Congo
